# Silver nanostructures synthesis via optically induced electrochemical deposition

**DOI:** 10.1038/srep28035

**Published:** 2016-06-13

**Authors:** Pan Li, Na Liu, Haibo Yu, Feifei Wang, Lianqing Liu, Gwo-Bin Lee, Yuechao Wang, Wen Jung Li

**Affiliations:** 1 State Key Laboratory of Robotics, Shenyang Institute of Automation, Chinese Academy of Sciences, Shenyang 110016, China; 2 University of Chinese Academy of Sciences, Beijing 100049, China; 3 School of Mechatronics Engineering and Automation, Shanghai University, Shanghai 200072, China; 4 Department of Power Mechanical Engineering, National Tsing Hua University, Hsinchu, Taiwan; 5 Department of Mechanical and Biomedical Engineering, City University of Hong Kong, Kowloon Tong, Hong Kong

## Abstract

We present a new digitally controlled, optically induced electrochemical deposition (OED) method for fabricating silver nanostructures. Projected light patterns were used to induce an electrochemical reaction in a specialized sandwich-like microfluidic device composed of one indium tin oxide (ITO) glass electrode and an optically sensitive-layer-covered ITO electrode. Silver polyhedral nanoparticles, triangular and hexagonal nanoplates, and nanobelts were controllably synthesized in specific positions at which projected light was illuminated. The silver nanobelts had rectangular cross-sections with an average width of 300 nm and an average thickness of 100 nm. By controlling the applied voltage, frequency, and time, different silver nanostructure morphologies were obtained. Based on the classic electric double-layer theory, a dynamic process of reduction and crystallization can be described in terms of three phases. Because it is template- and surfactant-free, the digitally controlled OED method facilitates the easy, low cost, efficient, and flexible synthesis of functional silver nanostructures, especially quasi-one-dimensional nanobelts.

Compared with their bulk counterparts, nanostructured materials (NSMs) are fascinating topics of research because of their size-dependent novel physical, chemical, optical, and magnetic properties^1^. The dimensionality of NSMs and its influence on their applications have been fully studied^2^, especially one-dimensional (1D) nanostructures^3^,^4^. Besides dimensionality, morphology is another important factor that influences the unique properties of NSMs^5^,^6^. Metallic nanostructures with fine size- and shape-controllability show great potential applications in electronic nanodevices, biological diagnostics, and surface-enhanced Raman scattering (SERS) detection^7^,^8^,^9^. For example, the controllability of the dimensions and aspect ratios of one-dimensional silver nanostructures such as nanowires and nanorods has been extensively studied for applications in flexible conductors and sensors^10^,^11^,^12^,^13^. Nanobelts, or nanoribbons, as quasi-one-dimensional nanostructures, were first proposed for semiconducting oxides^14^, and have attracted extensive attention for those substrates^15^ as well as various metal materials^16^,^17^ because of their well-defined geometrical morphologies with rectangular cross-sections, potential applications in bio-nanodevices^18^,^19^, and better inherent high-temperature stability^20^.

To date, a wealth of methods is available for generating silver nanobelts, including template-directed methods, seed-mediated methods, galvanic-cell reaction approaches, and electrochemical reduction methods. Liu *et al*. presented an electrochemical silver mirror reaction in anodic aluminum oxide (AAO) nanochannel templates to grow silver nanobelts with dimensions that depended on the AAO template^21^. It has been demonstrated that large-scale ultralong silver nanobelts can be synthesized at the interface between dibutyl sebacate (DBS) and silver nitrate solutions, which serves as a soft template, via a chemical electro-deposition technique^22^. By using biomass-derived monolithic activated carbon (MAC) as a template, Zhao *et al*. reported a galvanic-cell reaction method to obtain silver nanobelts with widths of 1.09 μm and a thickness of 0.29 μm, in high yield^23^. Recently, they improved the method by forming metallic particles on the MAC surface as growth initiators prior to nanobelt growth, and harvested silver nanobelts with widths as low as tens of nanometers and thicknesses of *ca.* 13 nm^24^. Zhu *et al*. reported an electrochemically modulated reduction method using a sacrificial cathode electrode with a nanochannel template to fabricate silver nanobelt-bundle arrays that were 25 nm thick, 300 nm wide, and 50 μm long^25^. These methods always require templates, or complex surfactants as capping agents, to guide the anisotropic quasi-1D nanobelt growth, which makes the fabrication process complex and inflexible. Additionally, external templates and agents have the potential to introduce impurities and contaminants into the final product. In this paper, we present a template- and surfactant-free method for producing different silver nanostructure morphologies, especially silver nanobelts with rectangular cross-sections, via optically induced electrochemical deposition (OED) in a microfluidic device. By virtue of an optically induced electrokinetics (OEK) platform^26^,^27^ previously developed by our group, we use programmable light patterns to produce controlled and localized electric fields in a photosensitive microfluidic chip, thus inducing the local electrochemical deposition of silver nanostructures. This new method has several advantages over traditional electrochemical reduction approaches: it offers a significant reduction in fabrication time (in terms of several seconds), is reagent-free (without the need for extra reductant or surfactant), and is template-free (with no lithographic process or complicated pre-elaboration). We have studied the parameters to controllably tune the final morphology of the silver nanostructures. We believe that this simple approach provides new opportunities to flexibly produce structured and functional nanomaterials for applications in fields such as photonics, biology, electronics, and other interdisciplinary areas.

## Methods

### Materials and OED system

**Figure 1:**
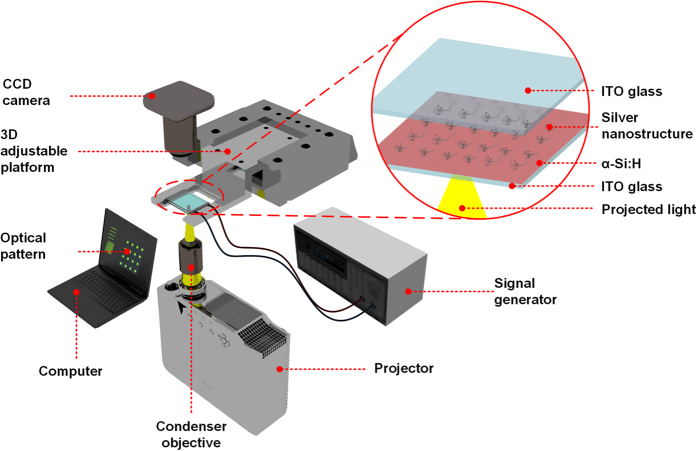
Schematic diagram of OED system. The enlarged circular area illustrates the structure of the OEK chip and the silver nanostructures (Ag NS) fabricated on the chip.

Silver nitrate (AgNO_3_, >99.8%; AR), purchased from Sigma Aldrich, was dissolved in Millipore water (resistivity, 18.2 MΩ, Millipore Systems) to prepare sample precursor solutions at concentrations of 10, 50, 100, and 200 mmol/L. The solutions were degassed in an ultrasonic cleaner at 40 kHz for 20 min (KUDOS, SK5210LHC) and further filtered through a 0.22 μm Millipore filter to remove undissolved solute. Figure 1 shows a schematic diagram of the experimental OED system, which includes three modules, as described in our earlier papers^26^,^27^. In brief, we use a computer equipped with graphics software to generate programmable light patterns on demand, which are projected on the sandwiched OEK microfluidic chip by an LCD projector (VPL-F400X, Sony, Japan) and an objective (CF Plan 50 X/0.55 EPI ELWD, Nikon, Japan). As shown in the enlarged circular area (Fig. 1), the sandwich-like OEK microfluidic chip, assembled from a top layer of indium tin oxide (ITO) glass and a bottom layer of ITO glass coated with a 1 μm film of hydrogenated amorphous silicon (α-Si:H) by the plasma-enhanced chemical vapor deposition (PECVD) method, is separated by a 60 μm spacer to form a reaction chamber. The α-Si:H film is a photoconductive layer which has a low dark conductivity of 10^−11^ S/m and an augmented illuminated conductivity of 10^−5^ S/m due to its photo-activated electron-hole pairs^28^. The entire microfluidic module is mounted on a three-dimensional adjustable platform for automatic motion control, monitored by a charge-coupled device camera (CCD, DaHeng Image DH-SV1411FC, China), and the alternating current (AC) voltage is applied by a signal generator (Agilent 33522A, USA).

### Preparation and characterization of silver nanostructures

The formulated silver precursors with different concentrations were injected into the OEK chip and powered by a sinusoidal AC voltage with a frequency and magnitude in the range of 1 to 100 kHz and 2 to 15 V_pp_, respectively. After the fabrication process, the prepared silver nanostructures were placed on the substrate, gently rinsed with anhydrous ethanol, and air dried at room temperature. Morphology observation was performed on a scanning electron microscope (SEM, Zeiss EVO MA, Germany). Dimensional data were characterized and collected via atomic force microscopy (AFM, Bruker Dimensional Icon, USA).

## Results and Discussion

### Virtual electrode-solution interface process

**Figure 2:**
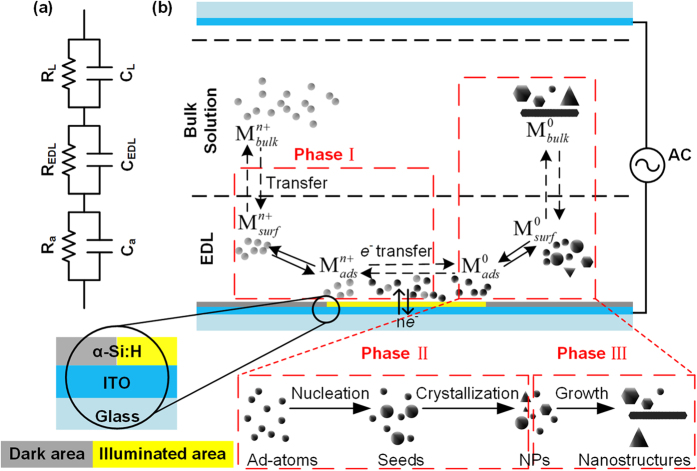
The equivalent circuit model of the OEK chip and OED process of silver nanostructure deposition. (**a**) Equivalent circuit of the series combination of α-Si:H virtual electrode impedance, EDL impedance, and the solution impedance. The EDL impedance is composed of a parallel combination of the capacitance,C_EDL_, and the resistance, R_EDL_, both of which are frequency-dependent. (**b**) Phase I describes the transfer of oxidized ions from the bulk solution 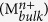 to the EDL surface 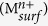 close to the virtual electrode to form adsorbed ions 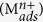, the subsequent electrochemical reaction to form the reduced adsorbed atoms 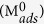, and the dynamic process of electron transfer. Phase II describes the nucleation and primary crystallization processes for the reduced ad-atoms 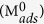 from seeds to nanoparticles (NPs). Phase III describes the crystal growth of the primary crystal nanostructures. The enlarged circular area shows the detailed structure of the bottom α-Si:H substrate. This figure has been enlarged in one direction for clarity.

In an electrochemical reduction system, the kinetics at the interface between the electrode and bulk solution plays a pivotal role in the formation of the final products^29^,^30^. In our OED system, at the interface between the illuminated solid α-Si:H substrate (namely, the virtual electrode) and bulk solution, a thin electric double layer (EDL, typically several nanometers) provides the voltage potential, reactive species, and environment for the overall reaction. Figure 2(a) shows a simplified equivalent circuit model of the OEK chip, where *C*_a_ and *R*_a_ denote the capacitance and resistance of the α-Si:H layer, *C*_L_ and *R*_L_ denote the capacitance and resistance of the bulk solution, and *C*_EDL_ and *R*_EDL_ indicate the equivalent capacitance and resistance of the EDL between the solid electrode and bulk solution, respectively, which vary depending on the applied voltage. The OED process described in Fig. 2(b) can be divided into three phases. Phase I represents the reduction of silver ions (Ag(I)) to elemental silver atoms (Ag(0)). This stage includes the mass transfer of Ag(I) from the bulk solution to the electrode-solution interface, and the electrochemical reduction of Ag(I) into Ag(0) at the EDL layer close to the virtual electrode under the effects of dynamic electron transfer. Phase II encompasses the nucleation and growth of the reduced silver atoms to form nuclei, seeds, and primary silver crystal structures. These silver atoms are transferred to the illuminated substrate and interact with each other, aggregating into small clusters. The clusters initially formed by the random collisions of the reduced silver atoms are too small to be stable, and are re-dissolved into the precursor solution. With continuous reduction, oversaturation occurs, and at a certain point defined by the nucleation energy barrier, the clusters grow beyond a critical size to form stable nuclei. These nuclei will serve as crystallization seeds for subsequent deposition. We are able to control the external applied voltage parameters to tune the reduction speed and kinetically control the nucleation process. Phase III depicts the crystal growth stage, in which the final silver nanostructures are formed. The growth process is a complex thermodynamically and kinetically controlled process that cannot be clearly divided^31^. When the reduction speed is fast, nuclei are formed in large quantity, which suppresses the continuing growth of the already formed nucleation sites. As a result, the final silver deposition products are homogenous small nanoparticles. When the reduction speed is relatively slow, the subsequent reduced silver atoms are deposited on the previously formed seeds (nucleation sites) and crystal growth proceeds, which is energetically favorable. The primary crystal seeds included silver nanospheres, polyhedral nanoparticles, and triangular or hexagonal nanoplates. As the seeding and growth continued, the final nanostructures included larger polyhedra, nanoplates, and nanobelts.

### Optimal parameters for synthesis of silver nanostructures

We examined the influence of the external parameters on silver deposition in the OED system. The concentration of the silver nitrate precursor solution was important for the formation of silver nanostructures. For the concentration series 10, 50, 100, and 200 mmol/L, larger precursor concentrations were found to increase the deposition speed, which is favorable for silver electrode deposition, as described in our previous paper^27^. On the other hand, the synthesis of silver nanobelts requires anisotropic control over the deposition process, which needs a relatively slow deposition speed, so the medium concentration of 50 mmol/L was selected for further experiments.

As the voltage was increased from 2 to 15 V_pp_, the silver reduction speed was increased. When the voltage was below 2 V_pp_, the current density in the EDL near the virtual electrode was insufficient for reduction of the Ag(I) into Ag(0). In contrast, when the voltage exceeded 15 V_pp_, the stronger voltage potential usually damaged the α-Si:H layer, which should preferably be avoided. The voltage range of 5 to 10 V_pp_ was selected as favorable.

**Figure 3:**
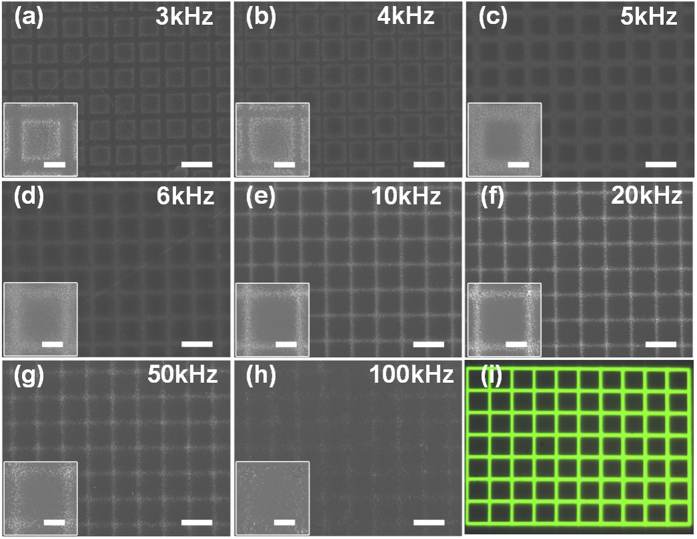
Silver deposition kinetics depends on the applied voltage frequency. (**a**–**h**) The silver deposition morphologies were dependent on the applied voltage frequency of 3, 4, 5, 6, 10, 20, 50, and 100 kHz, respectively. (**i**) The projected grid-shaped light pattern with green coloration reveals the illuminated area. The amplitude and time were set at 5 V_pp_ and 3 s, respectively, and the scale bars represent 20 μm. The insets show enlargements of the rectangular areas, and the scale bars represent 10 μm.

The frequency of the applied voltage is a key factor in the control of the final product’s nano-structural morphology, because it greatly influences the properties of the EDL. As shown in Fig. 3, we investigated the influence of the frequency on the deposition sites of the silver nanostructures. When the voltage frequency is below 10 kHz, the reduced silver nanostructures seem to be repelled from the grid-shaped illuminated virtual electrode area, and settle instead at the boundaries of the illuminated pattern. When the voltage frequency exceeds 10 kHz, deposition occurs mainly in the illuminated areas, in accordance with the defined virtual electrode. From our experimental results, a frequency between 20 and 50 kHz should be optimal for silver electrode deposition and will reproduce the designed light pattern with high fidelity. When the frequency is increased above 50 kHz but below 100 kHz, the silver deposition is somewhat sparse due to the reduced deposition cycle. In this range of frequency, the depositions tend to form anisotropic nanostructures such as thin silver nanosheets, triangular nanoplates and nanobelts.

**Figure 4:**
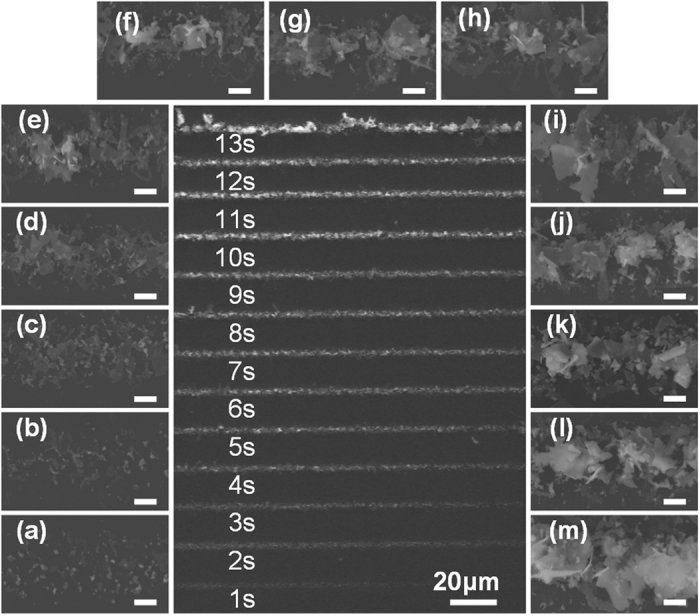
Time-dependent growth morphology during silver deposition. (**a**–**m**) show the detailed deposition densities based on times of 1–13 s, in sequence. The amplitude and frequency were 5 V_pp_ and 50 kHz, respectively. All scale bars represent 1 μm.

The deposition time was another important factor. The deposition thickness of the silver structures is positively correlated to the deposition time, at the proper frequency and amplitude of the applied voltage^27^. Here, we focused on the fabrication of silver nanobelts at a frequency of 50 kHz and amplitude of 5 V_pp_. Figure 4 shows a sequence of silver deposition images recorded at times from 1 to 13 s by SEM. Over time, the continuously reduced Ag(0) is adsorbed on the active sites of already formed primary seeds, crystallizes, and grows into tiny silver fragments, and then, thin silver sheets. These freshly formed silver thin sheets are potential sites for the subsequent growth of silver nanobelts, which can be found in Fig. 4(e–m).

### Morphologically controlled synthesis of silver nanostructures

**Figure 5:**
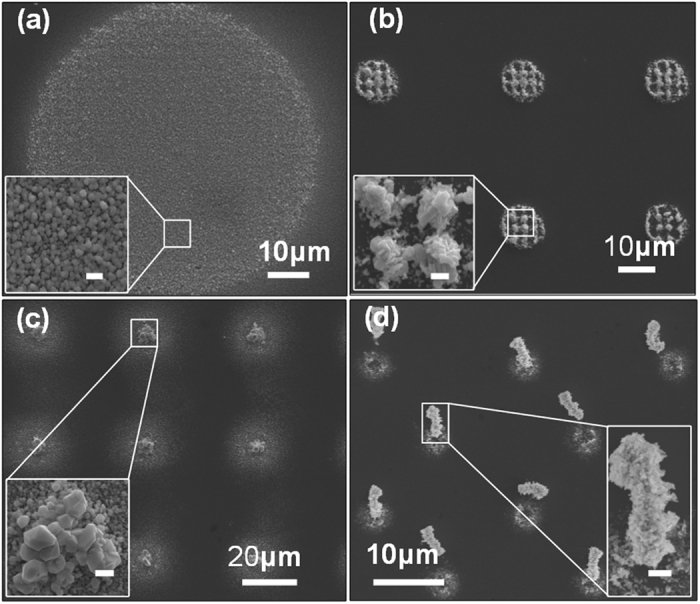
Primary silver crystallization structures under local electrochemical reaction defined by program-mable virtual electrodes. (**a**) Silver crystal nanoparticles with a solid circular virtual electrode. (**b**) Stacked silver hexagonal nanoplates with a crossed virtual electrode. (**c**) Crystallized silver octahedra and hexagonal silver nanoplates with focused dot-shaped virtual electrodes. (**d**) An array of silver nanoparticles assembled into dense micropillars with focused dot-shaped virtual electrodes. The delamination was caused by the post-rinsing process. The insets show the enlarged detailed morphologies of the silver deposits with different virtual electrodes. The deposits in images (**a**,**c**) were formed at voltage conditions of 20 kHz/5 V_pp_, whereas (**b**,**d**) were formed at 20 kHz/10 V_pp_. Inset scale bars in (**a**–**c**) represent 1  μm. Inset scale bar in (**d**) represents 2 μm.

Compared to traditional electrochemical systems, our OED system is sufficiently flexible and programmable for producing optically defined diverse virtual electrodes to control the growth of silver nanostructures. Figure 5 shows silver nanostructures deposited in different electrode configurations, which were defined by optical patterns. The large illuminated area of a solid circle in Fig. 5(a) resulted in massive polyhedral nanoparticles, whereas the relatively focused and reduced areas in Fig. 5(c) obtained larger polyhedral nanoparticles including octahedra and stacked hexagonal nanoplates under a voltage amplitude of 5 V_pp_. Figure 5(b,d) reveal denser and larger structures assembled from plate-like silver polyhedra under augmented voltage amplitudes of 10 V_pp_. Larger light pattern areas provide correspondingly larger virtual electrode areas which simultaneously increase the quantity of reduced silver atoms at the same solution concentration, and thus favor homogenous nucleation and the synthesis of small nanoparticles. With decreased virtual electrode areas triggered by focused light patterns, the deposited silver nanostructures tend to ripen and form larger nanostructures, such as silver octahedral nanoparticles and hexagonal nanoplates.

**Figure 6:**
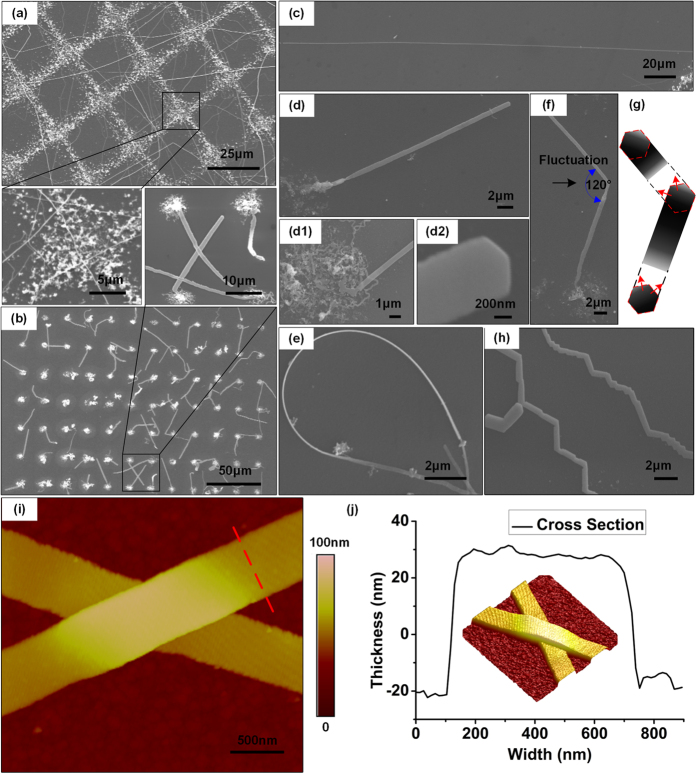
Crystal growth and morphological evolution of the primary silver deposits under local electrochemical reaction defined by programmable virtual electrodes. (**a**) Crossed and long silver nanobelts decorate a silver grid deposited over a grid-shaped virtual electrode. The enlarged area shows the crossing point and silver nanobelt origin point. (**b**) An array of single silver nanobelts extruded from a single dot deposited with a dot-shaped virtual electrode. The enlarged area shows the details. (**c**) The maximum length of the silver nanobelt was ca. 300 μm. (**d**) A single straight silver nanobelt originating from a silver dot deposit. (**d1**) and (**d2**) show the enlarged original point and terminal morphology of the single silver nanobelt in (**d**). (**e**) A curled silver nanobelt, which shows the material’s flexibility. (**f**) SEM image showing a single silver nanobelt with a 120° turn. (**g**) Possible growth mechanism of silver nanobelts along two neighboring edges of the primary silver hexagonal nanoplates. The nanobelts exhibit 120° turns in the growth direction under the possible fluctuation of conditions. (**h**) AFM image of two interconnected nanobelts. (**j**) Rectangular cross-section of a single nanobelt (indicated by the red dashed line in (**i**)) shows a width of 500 nm and thickness of 50 nm. The inset shows 3D AFM image of the nanobelts in (**i**).

To obtain silver nanobelts, we set the applied voltage at 50 kHz and 5 V_pp_, which is favorable for the synthesis of silver anisotropic nanostructures rather than compact silver electrodes. By kinetically controlling the deposition process, the reduced silver atoms were deposited on already formed seeds in phase II and crystallized into primary polyhedral nanostructures, rather than forming fresh nucleation sites and resulting in massive silver nanoparticles. Figure 6(a) displays a grid-shaped silver deposit decorated with interconnected silver nanobelts, and Fig. 6(b) presents an array of silver nanobelts stemming from dot-shaped silver deposits, which form a functional silver nanobelt network and probe array that could have potential applications in biosensors and nanodevices. These results show that silver nanobelts with different configurations can be obtained by simply tuning the projected light pattern, which is a flexible and time-saving approach. Figure 6(c) shows silver nanobelts as long as 300 μm that were fabricated by our method. Figure 6(d,e) show the details of the silver nanobelts. Figure 6(d1,d2) show close-ups of the stem and terminal tip of the silver nanobelt in Fig. 6(d), which demonstrate that the silver nanobelts originate from a sheet-like structure and end with a tip of 120°. Figure 6(e) shows a curled silver nanobelt, confirming its flexibility. Figure 6(f–h) show the supposed growth process for the silver nanobelts. The silver nanobelts are formed from 2D silver nanosheets in accord with a specific crystallographic orientation. Some silver nanobelts were found to alter their growth direction under dislocation fluctuations, forming silver nanobelts with zigzag shapes and a constant angle of 120°. This phenomenon implies that the silver nanobelts originate from the primary hexagonal nanoplates and grow along two adjacent edges to form the anisotropic belt-like structures. The AFM images of two crossed silver nanobelts in Fig. 6(i,j) show a smooth surface and a rectangular cross section, with a width of *ca.* 500 nm and thickness of *ca.* 50 nm.

## Conclusion

In summary, we have presented a flexible OED system for the controllable synthesis of silver nanostructures with various morphologies and configurations. By fine-tuning the applied voltage and deposition time, silver polyhedral nanoparticles, hexagonal silver nanoplates, and especially, silver nanobelts, were synthesized. The fabrication process is template-free and agentless. The obtained silver nanobelts were rectangular in cross-section with an average width of 300 nm, average thickness of 100 nm, and length of about 300 μm. A possible theory of silver nanobelt formation process is also discussed in this paper in terms of growth along two neighboring sides of a primary hexagonal silver nanoplate. We believe that the OED system described in this paper has the potential for use in the deposition of other metals as well as nanostructure synthesis with diverse functional configurations.

## Additional Information

**How to cite this article**: Li, P. *et al*. Silver nanostructures synthesis via optically induced electrochemical deposition. *Sci. Rep.* **6**, 28035; doi: 10.1038/srep28035 (2016).
